# Real-World-Time Data and RCT Synergy: Advancing Personalized Medicine and Sarcoma Care through Digital Innovation

**DOI:** 10.3390/cancers16142516

**Published:** 2024-07-11

**Authors:** Philip Heesen, Georg Schelling, Mirko Birbaumer, Ruben Jäger, Beata Bode, Gabriela Studer, Bruno Fuchs

**Affiliations:** 1 Faculty of Health Sciences & Medicine, University of Lucerne, Frohburgstrasse 3, 6002 Luzern, Switzerland; philip.heesen@uzh.ch (P.H.); gabriela.studer@luks.ch (G.S.); 2 Sarcoma Service, Department of Orthopedics and Trauma, Sarcoma Center, LUKS University Hospital, 6000 Lucerne, Switzerland; 3 Medical Faculty, University of Zurich, 8032 Zurich, Switzerland; 4 Lucerne University of Applied Sciences and Arts/HSLU, Werftestrasse 4, 6002 Luzern, Switzerland; mirko.birbaumer@hslu.ch; 5 Sarcoma Service, Klinik für Orthopädie und Traumatologie, Sarcoma Center, Kantonsspital Winterthur, 8400 Winterthur, Switzerland

**Keywords:** real-world/time data/evidence (RWTD/E), randomized controlled trials (RCTs), healthcare research, personalized medicine, population health management, sarcoma care, digital twin, target trial emulation, value-based healthcare (VBHC), patient outcome optimization

## Abstract

**Simple Summary:**

This study looks at how combining real-world/time data/evidence (RWTD/E) with traditional clinical studies (known as randomized controlled trials) can improve healthcare research and ultimately patient care. Unlike past methods that often rely on outdated information, RWTD/E provides up-to-the-minute data on patient health, making it a powerful tool for doctors and researchers. This approach is especially useful in areas like sarcoma, a type of cancer, where understanding each patient’s unique situation can lead to improved treatment plans. We discuss how this mix of real-world/time data and traditional research can lead to more personalized medicine, helping to create treatments tailored to individual patient needs. It also aids in managing health at a community level by spotting trends and improving health policies, making care more efficient and focused on what patients truly benefit from. Furthermore, we explore how applying new methods, like creating digital twins of patients’ health profiles to RWTD/E can help in predicting individualized treatment effects. This study aims to elaborate that using prospective RWTD/E alongside traditional clinical trials can make healthcare more effective, patient-focused, and ready to adapt to new discoveries and technologies.

**Abstract:**

This manuscript examines the synergistic potential of prospective real-world/time data/evidence (RWTD/E) and randomized controlled trials (RCTs) to enrich healthcare research and operational insights, with a particular focus on its impact within the sarcoma field. Through exploring RWTD/E’s capability to provide real-world/time, granular patient data, it offers an enriched perspective on healthcare outcomes and delivery, notably in the complex arena of sarcoma care. Highlighting the complementarity between RWTD/E’s expansive real-world/time scope and the structured environment of RCTs, this paper showcases their combined strength, which can help to foster advancements in personalized medicine and population health management, exemplified through the lens of sarcoma treatment. The manuscript further outlines methodological innovations such as target trial emulation and their significance in enhancing the precision and applicability of RWTD/E, underscoring the transformative potential of these advancements in sarcoma care and beyond. By advocating for the strategic incorporation of prospective RWTD/E into healthcare frameworks, it aims to create an evidence-driven ecosystem that significantly improves patient outcomes and healthcare efficiency, with sarcoma care serving as a pivotal domain for these developments.

## 1. Introduction

In the dynamic context of evidence-based healthcare, real-world/time data/evidence (RWTD/E) assumes a critical role, extending past the limitations of randomized controlled trials (RCTs) that might arise when randomization would be unethical or when an RCT would be too costly to undertake [1,2,3,4,5]. The progression towards a joint use of RCTs and RWTD/E signifies a fundamental paradigmatic transition towards the incorporation of exhaustive, contemporaneous patient data, augmenting the depth and breadth of healthcare research and decision-making mechanisms [6,7,8]. Emphasizing this evolution, the first part traces the trajectory from an exclusive reliance on conventional RCT methodologies to a comprehensive, data-informed strategy. The integration of RWTD/E with RCTs represents a significant advancement in evidence-based healthcare [1]. RWTD/E’s unique ability to provide real-world/time, contextual data offers a complementary perspective to the controlled environments of RCTs. This synthesis enhances our understanding of healthcare interventions by bridging the gap between clinical trial settings and real-world/time patient experiences, thereby enriching the evidence base for more informed healthcare decisions and policies [9].

RWTD/E is not only an important complimentary part to RCTs, but also contrasts sharply with traditional retrospective analyses of health data (Table 1). RWTD/E enhances the granularity and relevance of healthcare data, providing immediate insights into treatments and outcomes prospectively. This evolution not only captures the fluidity of healthcare interactions but also offers substantial advantages over retrospective data by enabling timely, informed decision-making and a deeper understanding of patient care processes [10].

This study’s strategic importance lies in its potential to significantly influence the ongoing evolution of healthcare research, quality, and policy [11]. By integrating prospective RWTD/E with RCTs, it aims to bridge the gap between clinical evidence and practical application, thus enhancing patient outcomes and informing evidence-based policy decisions. This manuscript contributes to critical debates on optimizing healthcare delivery through a nuanced understanding of real-world/time patient experiences and outcomes, positioning itself at the forefront of advancing healthcare effectiveness and efficiency [12,13,14]. Therefore, we herein explore the potential and synergistic value of prospective RWTD/E alongside RCTs in enriching healthcare research and decision-making processes.

## 2. Real-World/Time Data/Evidence: Foundations and Future

RWTD/E encompasses the prospective collection and analysis of healthcare data as it occurs, capturing the continuum of patient care also known as the patient pathway, in real-world and real-time [1,3]. A patient pathway delineates the comprehensive journey of a patient through the healthcare system for specific conditions or treatments, highlighting the sequence of care events and interactions with healthcare providers. This concept is paramount for the future as it underscores the importance of understanding and optimizing the entire spectrum of patient care, from initial consultation through treatment and follow-up, thereby enhancing patient outcomes and potentially aligning with value-based healthcare (VBHC) principles by enabling the accurate assessment of care cycle costs [15]. This approach includes data from electronic health records, patient-generated data, and other healthcare interactions, providing a comprehensive view that reflects the temporal and contextual realities patients face across various settings [16]. RWTD/E’s defining feature is its ability to offer a dynamic, ongoing snapshot of healthcare processes, setting a foundation for evidence that is both immediate and reflective of the complexities of real-world patient experiences.

Prospective RWTD/E and RCTs serve distinct, complementary roles in evidence generation [17]. RCTs, with their structured and controlled setup, are designed to minimize bias and establish causality under specific conditions. In contrast, RWTD/E offers a broader, more inclusive approach by capturing data in real-life settings, reflecting the complexity and diversity of patient experiences. This expansive nature of RWTD/E complements the precision of RCTs, providing a more holistic understanding of healthcare interventions across different populations and settings.

The scope of RWTD/E spans from electronic health records to patient-generated data, encompassing a wide array of healthcare interactions. This breadth allows RWTD/E to provide invaluable insights across diverse healthcare scenarios, reflecting the real-life complexity and variability of patient care. Through the aggregation and analysis of this extensive data, RWTD/E enhances our understanding of healthcare outcomes, treatment effectiveness, and patient experiences in various settings, underscoring its critical role in advancing evidence-based medicine.

Prospective RWTD/E provides distinct advantages over retrospective data by dynamically capturing and analyzing healthcare events as they occur. This immediacy equips RWTD/E to mirror the current state of patient care accurately, yielding real-world/time insights into treatment outcomes and healthcare delivery effectiveness. The inherent dynamism of RWTD/E supports a healthcare system that is both responsive and adaptable, swiftly integrating the latest evidence into patient care pathways. Conversely, retrospective data, while offering historical insights, is static and grounded in past healthcare practices. It is subject to significant selection biases, as it often relies on pre-existing records that may not represent the full spectrum of patient experiences. Such biases limit the ability to generalize findings to broader patient populations. The temporal aspect of RWTD/E signifies its unique advantage, offering continuous data collection that provides a dynamic, evolving picture of patient care and outcomes. This continuous timeline of data captures the nuances of healthcare processes in real time, enabling a deeper, more accurate understanding of treatments, patient responses, and the effectiveness of healthcare interventions as they unfold.

RWTD/E demonstrates remarkable adaptability and relevance across diverse healthcare settings, from hospitals and clinics to remote patient monitoring and home care. Its application spans the healthcare continuum (“patient pathway”), offering insights into patient care processes and system efficiencies in real-world/time. This versatility supports personalized patient care strategies, public health initiatives, and health policy development, showcasing RWTD/E’s essential role in advancing healthcare delivery and outcomes across various environments.

RWTD/E enhances the insights gained from RCTs by providing a broader understanding of treatment effectiveness and patient experiences in real-time settings. This complementary relationship allows for a more comprehensive evaluation of healthcare interventions, filling gaps in knowledge about their real-world applicability and impact on patient-centered outcomes.

The future of RWTD/E looks promising, with anticipated technological innovations and advancements in data analytics expected to significantly enhance its utility and impact. These developments promise to deepen our understanding of healthcare processes, improve patient outcomes, and drive more informed decision-making across the healthcare spectrum.

## 3. Enhancing Healthcare Insights through Prospective RWTD/E

RWTD/E offers a nuanced perspective on patient outcomes, capturing the diverse experiences and journeys of patients (“patient pathways”) across healthcare settings. This approach is vital for benchmarking between institutions, both nationally and internationally, especially using quality indicators for specific care areas like sarcoma. By examining data from varied healthcare experiences, RWTD/E enables a broader understanding of patient outcomes, supporting the development of more effective healthcare strategies and policies through benchmarking efforts [18,19]. This enhanced view is essential for advancing patient care and ensuring high-quality healthcare delivery across different regions and populations.

RWTD/E captures a wide spectrum of patient demographics, including those often underrepresented in traditional healthcare research. This comprehensive data collection enables a deeper understanding of treatment effects across diverse populations [20,21]. Sarcoma care guidelines, for instance, show a variety of treatment pathways, indicating that patient care can significantly vary not only within a single country but also across different geographical regions. RWTD/E’s expansive dataset offers critical insights into these variations, emphasizing the need for personalized treatment strategies that consider the unique contexts of different patient groups [22,23].

RWTD/E can offer valuable insights beyond what is typically captured in RCTs, especially regarding drug effectiveness and patient adherence [2]. For sarcoma, where individual patient responses can vary widely due to the heterogeneity of the disease, RWTD/E can highlight how treatments perform in a broader, more diverse population, potentially revealing variations in effectiveness and adherence not apparent in the controlled conditions of RCTs. This underscores the importance of integrating RWTD/E for a more nuanced understanding of treatment outcomes in sarcoma care.

RWTD/E embraces the intricacies of healthcare in real-world/time settings, recognizing factors like comorbidities and diverse healthcare practices that RCTs often overlook. This comprehensive approach allows for a more authentic understanding of how treatments work in the complex web of patient care, highlighting the need for flexible, adaptable healthcare strategies that can accommodate the full spectrum of patient experiences.

Among the illustrative case studies demonstrating RWTD/E’s substantial contributions to healthcare insights, a notable example is the benchmarking study comparing two multidisciplinary teams (MDTs) in sarcoma treatment [18]. Utilizing the interoperable digital platform Sarconnector^®^ for real-world/time data assessment (which is currently accessible through www.adjumed.net), this study unveiled significant variations in treatment approaches, particularly in chemotherapy indications, but also in indications for biopsies or surgeries, among other areas [18]. Such discrepancies underscore the critical importance of RWTD/E in identifying and addressing gaps in care delivery. This benchmarking effort not only highlights RWTD/E’s capability to standardize and evaluate quality and cost metrics across different healthcare settings, but also emphasizes its potential to drive data-driven optimizations in patient care strategies. Through the harmonized data approach facilitated by Sarconnector^®^ (assessing, for example, a minimal data set (Appendix A), healthcare providers can pinpoint areas for improvement, leading to enhanced patient outcomes and more efficient healthcare delivery. This case exemplifies RWTD/E’s power in advancing a value-based care model by providing actionable insights that are pivotal for improving healthcare quality, efficiency, and patient satisfaction across diverse medical disciplines beyond sarcoma care [24].

Additionally, the application of RWTD/E can lead to more cost-efficient healthcare delivery by identifying the most effective treatments and avoiding unnecessary or less effective interventions [25].

Emulating target trials with RWTD/E represents a pivotal advancement in harmonizing the rigorous methodologies of RCTs with the intricate realities of real-world/time healthcare [26,27,28]. This sophisticated approach capitalizes on the comprehensive datasets afforded by RWTD/E to approximate the conditions and controls of RCTs, thereby offering a refined method for assessing intervention outcomes among diverse patient populations. Such an emulation not only corroborates and broadens the applicability of clinical trial findings, but also fosters the tailoring of treatment strategies to meet the individual needs and circumstances of patients. Consequently, it plays a vital role in the evolution of personalized medicine, enabling healthcare providers to devise more effective and flexible care plans that reflect the genuine complexities of patient experiences. In doing so, target trial emulation underscores the transformative potential of RWTD/E in elevating healthcare quality, efficiency, and patient satisfaction, thereby reinforcing its indispensable contribution to advancing a value-based care paradigm. 

## 4. Methodological Advancements in RWTD/E

Advancements in data capture technologies, such as wearable devices and mobile health applications, have significantly propelled the capabilities of RWTD/E [29,30,31]. These innovations allow for the continuous, real-world/time monitoring of patient health metrics and behaviors, providing a wealth of data that are both granular and expansive. This evolution in data collection not only enriches the depth of RWTD/E but also broadens its applicability across healthcare research, enhancing our understanding of patient experiences and outcomes in everyday life.

The integration of advanced analytical techniques, such as machine learning and artificial intelligence (AI), has been transformative in processing complex RWTD/E datasets [32,33,34,35] and might enable the accurate prediction of outcomes or individualized treatment effects and eventually the optimal choice of a therapy sequence for an individual patient. 

The interpretation of RWTD/E is being significantly enhanced through the use of sophisticated visualization tools and the fostering of cross-disciplinary collaborations. These strategies improve the comprehensibility and actionable nature of complex datasets, allowing for a more nuanced understanding across various healthcare domains.

Digital health platforms play a crucial role in prospective RWTD/E by aggregating data from diverse sources, ensuring consistency, and enabling comprehensive analyses [36]. These platforms, enhanced by AI, improve data collection, analysis, and interpretation, which are pivotal for patient-centered models like the ‘Sarcoma Digital Twin’ [31,37,38]. This concept represents an innovative approach to sarcoma care, creating detailed, personalized models that reflect individual patient conditions and treatment responses, facilitating better healthcare decisions.

Efforts to enhance the quality and reliability of RWTD/E are further complemented by implementing standardization initiatives and conducting validation studies, ensuring that data collected from diverse sources remain consistent and accurate. Alongside these critical efforts, the emulation of target trials emerges as a pivotal strategy, bolstering the credibility and applicability of RWTD/E findings. This approach meticulously simulates the controlled conditions of RCTs within the RWTD/E framework, employing rigorous analytical methods to ensure that real-world/time evidence maintains a high level of precision and relevance. By integrating emulated target trials into the standardization and validation process, RWTD/E not only adheres to the highest standards of data integrity but also significantly advances the utility of real-world/time insights in clinical research and healthcare decision-making. Such methodological sophistication highlights the transformative potential of RWTD/E, positioning it as an indispensable resource in the evolution towards more evidence-driven, patient-centered healthcare models.

Methodological advancements in RWTD/E, notably through the integration of target trial emulation, have significantly transformed the landscape of real-time patient monitoring and intervention. This methodology enhances the capacity for immediate and impactful healthcare interventions, directly contributing to the optimization of patient care practices and the achievement of superior health outcomes. The implementation of RWTD/E, enriched by the principles of target trial emulation, heralds a new era in patient care—one that is deeply rooted in evidence-based, personalized treatment paradigms and geared towards maximizing patient well-being and satisfaction. Through this innovative lens, RWTD/E is set to redefine expectations for treatment efficacy and patient care standards, demonstrating a commitment to advancing healthcare through methodological excellence and technological integration.

## 5. RWTD/E in Informing Healthcare Policy and Practice

In the context of modern healthcare policy development, real-world/time data/evidence (RWTD/E) is instrumental in underpinning the creation and refinement of evidence-based guidelines and protocols. RWTD/E facilitates a data-driven policymaking process by providing empirical insights into the effectiveness, safety, and patient satisfaction of healthcare interventions. Such evidence ensures that healthcare policies are reflective of the current clinical practices and patient needs, enabling policy frameworks to be dynamically adjusted in response to the evolving healthcare landscape. Consequently, RWTD/E serves as a critical resource for policymakers, guiding the establishment of healthcare standards that are both efficacious and patient-centric. This approach not only optimizes healthcare delivery but also ensures that policy adaptations are continuously informed by comprehensive, real-world/time evidence, thereby promoting better health outcomes and system efficiency.

RWTD/E may exert a profound impact on the formulation of clinical guidelines by furnishing a rich tapestry of data that encapsulate patient outcomes and the efficacy of treatments within real-world contexts. The analytic process of dissecting RWTD/E to unveil underlying patterns and trends offers a comprehensive perspective that transcends the confines of traditional controlled trials. Such a breadth of evidence empowers guideline developers to integrate a more diverse and representative array of findings, ensuring that clinical guidelines are not only grounded in empirical reality but are also finely tuned to the multifaceted nature of patient care. Consequently, the incorporation of RWTD/E into guideline development translates to protocols that are inherently aligned with actual clinical practices and patient experiences, fostering enhancements in health outcomes and elevating patient satisfaction through guidelines that are both responsive and reflective of real-world/time healthcare dynamics.

RWTD/E critically elevates healthcare decision-making by supplying real-time, evidence-based insights crucial for shaping both institutional strategies and national healthcare policies. This data-driven paradigm enables healthcare executives and policymakers to enact decisions deeply attuned to the evolving needs of patients and the prevailing healthcare environment. By integrating RWTD/E, decision-makers are equipped with a dynamic toolset to navigate and respond to the complexities of healthcare delivery, ensuring that interventions and policies are not only informed by the latest evidence but are also precisely targeted to enhance efficiency and effectiveness within the healthcare system. Thus, RWTD/E stands as a pivotal asset in the advancement of healthcare strategies, promoting a more informed, agile, and patient-centric approach to healthcare governance.

RWTD/E fundamentally undergirds the tenets of value-based healthcare by furnishing granular insights into the efficacy and economic efficiency of healthcare interventions from a real-world/time perspective. This methodology facilitates a nuanced evaluation of interventions, emphasizing their tangible impact on patient health outcomes alongside their cost implications. By leveraging RWTD/E, healthcare decision-makers are empowered to conduct comprehensive assessments that foreground the value delivered to patients and the system at large, enabling a judicious allocation of resources towards interventions that demonstrably maximize clinical benefit while optimizing economic utilization [25]. Consequently, RWTD/E serves as an essential instrument in the operationalization of VBHC, driving the transition towards healthcare models that prioritize measurable improvements in patient outcomes and sustainability of healthcare provisioning.

RWTD/E plays a critical role in augmenting healthcare quality and patient safety by facilitating the identification of improvement opportunities via the analysis of real-world/time data. This analytical exploration into RWTD/E uncovers actionable insights into patient care dynamics, revealing efficacy gaps and safety concerns that might not be apparent within the confines of controlled research environments. Such revelations often catalyze significant policy reformulations, ensuring that clinical practices and protocols are meticulously aligned with contemporary evidence delineating optimal care strategies. Consequently, the iterative refinement of healthcare delivery, informed by RWTD/E, underscores a commitment to elevating the standards of patient care, enhancing safety protocols, and fostering a healthcare ecosystem where quality and safety are perpetually optimized based on real-world patient outcomes and experiences.

The effective utilization of real-world/time data/evidence necessitates the active engagement of a diverse consortium of stakeholders, encompassing healthcare providers, patients, policymakers, and researchers [11]. This multidisciplinary collaboration is pivotal for the holistic integration of RWTD/E into healthcare policy and practice, ensuring that derived insights are comprehensively leveraged to inform and enhance healthcare delivery systems. The synergistic involvement of these stakeholders facilitates a multifaceted perspective in the analysis and application of RWTD/E, fostering policies and practices that are not only evidence-based but also reflect the collective expertise and experiences of the entire healthcare ecosystem. Consequently, this collaborative model amplifies the impact of RWTD/E on healthcare outcomes, driving forward improvements that are both informed by robust data and aligned with the nuanced needs and expectations of all parties involved. Such concerted efforts underscore the importance of stakeholder engagement in maximizing the potential of RWTD/E for substantial and sustainable healthcare advancements.

The advent of digital health and artificial intelligence (AI) marks a fundamental transformation in healthcare, steering it towards more value-based models [18,37]. This shift, catalyzed by the strategic integration of real-world/time data/evidence, is fundamentally altering the landscape of healthcare policy, the development of clinical guidelines, and the dynamics of stakeholder engagement. Recent studies elucidate the transformative potential of digital health and AI in crafting a healthcare ecosystem that is not only more efficient and equitable but also deeply rooted in patient-centric care. For instance, digital health tools and AI have been shown to shift healthcare from reactive to proactive by predicting and preventing diseases, as demonstrated in the transformation of health systems in low- and middle-income countries [39]. For example, in Kenya, the Leap platform uses AI-powered mobile health solutions to train community health workers to manage chronic diseases, leading to early interventions and improved health outcomes [39]. Additionally, AI enhances diagnostic accuracy, personalized treatment plans, and proactive disease prevention, further emphasizing its role in improving patient outcomes and operational efficiency [40,41]. By harnessing RWTD/E, this paradigmatic shift in healthcare is underscored, showcasing a move towards policy formulations and clinical practices informed by real-world/time, granular insights and outcomes. This evolution reflects a commitment to leveraging cutting-edge technology and comprehensive data analytics to refine healthcare outcomes and operational efficiency, ultimately guiding the transition towards a healthcare system that prioritizes value-based models. In this context, RWTD/E emerges as a pivotal force in driving informed decision-making and fostering an environment where healthcare delivery is continuously optimized to meet the intricate needs of patients, thereby reinforcing the strategic importance of digital health and AI in realizing a future characterized by informed, efficient, and patient-centered healthcare.

## 6. Case Studies and Practical Applications of RWTD/E

Diverse case studies from the Swiss Sarcoma Network (SSN), such as leveraging the Sarconnector^®^ platform, have illuminated the potential transformative impact of real-world/time data/evidence (RWTD/E) on patient outcomes in sarcoma care [18,19,37]. For example, these studies show that the use of real-world/time data analysis for benchmarking of the diagnostic pathway analysis has led to improved adherence to clinical guidelines, optimized treatment protocols, and enhanced patient outcomes, demonstrating the critical role of continuous quality improvement in sarcoma treatment [20]. These initiatives facilitate multidisciplinary team decision-making processes for complex sarcoma cases, enabling the exploration of integrating patient-specific data, real-world/time analysis, and predictive modeling. Such efforts lay the groundwork for personalized treatment plans that promise to markedly improve patient outcomes, highlighting RWTD/E’s broader application in tailoring treatments across various healthcare settings.

In advancing toward personalized medicine, the SSN is exploring the potential of prospective RWTD/E through initiatives like Digital Twinning and Predictive Analytics (DTPA), aiming to stand at the forefront of this innovation [31,37,38]. By laying the foundation for creating digital twins of patients’ medical profiles, the SSN envisions enabling tailored treatment strategies that account for the individual variability in disease progression and response to therapy. This forward-looking approach, intended to leverage RWTD/E to analyze patient outcomes and genetic information, underscores the envisioned pivotal role of RWTD/E in optimizing patient care and improving outcomes, embodying the future of personalized treatment plans.

The Swiss Sarcoma Network’s prospective RWTD/E Data Warehouse/-Lake exemplifies the strategic vision for utilization in population health management by offering a proposed comprehensive view of health trends and patient outcomes for sarcoma [18]. This vision aims to support the identification and analysis of health trends, facilitating the development of targeted preventive strategies and effective resource allocation. By planning to harness RWTD/E, stakeholders anticipate enhancing public health responses and tailoring health initiatives to diverse community needs, showcasing RWTD/E’s potential revolutionary impact on population health management [25].

The planned integration of RWTD/E into the SSN’s clinical workflows, particularly through initiatives like the weekly MDT/SB Tumor Conference, aims to demonstrate the practical benefits of real-world/time data in clinical decision-making. This collaborative enhancement of patient care pathways reflects RWTD/E’s wider potential for integration across healthcare settings, with ambitions to identify effective treatments, reduce hospital readmission rates, and enhance patient management practices, significantly contributing to healthcare delivery optimization and patient care across disciplines.

The SSN’s envisioned Quality Metrics and Outcome Measures initiative is anticipated to underscore RWTD/E’s impact in driving measurable improvements in healthcare quality and patient safety (Appendix A). Through the future systematic evaluation of sarcoma care effectiveness and efficiency, informed by real-world/time data, the SSN aims to implement policy adjustments and practice enhancements. These projected data-driven advancements reflect RWTD/E’s transformative potential in optimizing healthcare delivery and improving patient outcomes, demonstrating its wide-ranging impact across multiple healthcare domains.

## 7. Future Directions

As we navigate the evolving landscape of healthcare, the anticipatory advancements in RWTD/E herald a new era of precision and personalization in patient care. Emerging technologies, notably AI-driven analytics and blockchain for secure, ethical data sharing, are set to further refine RWTD/E’s capabilities [33,35,42]. These innovations promise to enrich healthcare insights, offering unprecedented accuracy and predictability in treatment outcomes and healthcare strategies. Such technological progress underscores RWTD/E’s pivotal role, as demonstrated by the Swiss Sarcoma Network (SSN) initiatives, in bridging the gap between clinical evidence and its practical application, enhancing patient outcomes, and informing policy with the depth of real-world/time insights.

The integration of prospective RWTD/E with diverse data sources, including IoT devices and genomics, represents a comprehensive approach to patient health analysis [42]. This multidimensional perspective—encompassing medical, lifestyle, and environmental factors—will advance the precision medicine frontier, tailoring healthcare delivery to individual needs while facilitating broader, population-wide health evaluations. This envisioned integration aligns with the SSN’s prospective strategies, aiming to leverage RWTD/E for a holistic understanding and management of sarcoma care, reflecting a broader applicability across healthcare disciplines.

Addressing future challenges, particularly around data privacy, standardization, and interoperability, remains paramount as RWTD/E expands. Ensuring the ethical use of patient data, while promoting research collaboration, echoes the discussions in previous chapters on the necessity for a balanced approach to data sharing that respects privacy and maximizes healthcare innovation [43,44,45]. Efforts towards developing universal data standards and employing interoperable platforms will be critical in realizing the full potential of RWTD/E, enabling seamless integration and efficient data utilization across the healthcare continuum [46,47,48].

Furthermore, enhancing patient engagement through prospective RWTD/E, as envisioned by initiatives like Sarconnector^®^, presents an opportunity to revolutionize the patient-provider dynamic. Enabling real-world/time access to health data and care plans empowers patients to actively participate in their healthcare journey, contributing to research and shared decision-making processes [35,49]. This direct involvement is poised to foster a more collaborative, informed, and patient-centered healthcare experience, highlighting the importance of RWTD/E in facilitating meaningful patient engagement and personalized care.

## 8. Conclusions

In conclusion, the strategic importance of prospective RWTD/E in advancing healthcare research, practice, and policy is undeniable. It stands as a testament to the power of integrating comprehensive, real-world/time data into healthcare decision-making, offering a path to more effective, efficient, and patient-centered care. As we reflect on the insights and advancements discussed throughout this manuscript, it is clear that the journey of RWTD/E is only beginning. Its continued evolution will require not only technological innovation but also a commitment to ethical practices and multidisciplinary collaboration. The Swiss Sarcoma Network’s ongoing efforts exemplify this journey, charting a course toward a future where healthcare is transformed by the depth and dynamism of real-world/time data/evidence.

## Figures and Tables

**Table 1:** Overview of data types in healthcare research: definitions, sources, and contexts of use.

Term	Definition	Source of Data	Timing of Data Collection	Usage Context
Retrospective data	Data collected from past events, often through examination of existing records such as medical charts and billing information	Historical records, medical charts, insurance claims	Collected after all events have occurred, looking back in time	Analyzing past events, to understand outcomes, trends, and areas for future research. Subject to selection biases
Prospective data	Data collected from the initiation of the study forward, with specific research questions and data collection processes in mind	Direct observations, surveys, clinical assessments.	Collected moving forward from the start of the study	Observing and assessing outcomes following specific exposures or interventions, minimizing recall biases and clarifying temporal relationships
Randomized controlled trials (RCTs)	Health-related data derived from sources outside traditional clinical trials, including EHRs, registries, and patient generated data	Controlled experimental settings	Prospective, starting at the time of the trial	Establishing causality between interventions and outcomes, minimizing bias through randomization and control
Real-world data (RWD)	Health-related data derived from sources outside traditional clinical trials, including EHRs, registries, and patient generated data	EHRs, claims, registries, patient devices	Not specific to real time; collected during routine clinical practice	Capturing patient experiences in routine practice to understand healthcare delivery and outcomes in natural settings
Real-world evidence (RWE)	Clinical evidence regarding the usage and potential benefits or risks of a medical product derived from analyzing RWD	Derived from RWD	Analysis occurs at any time post data collection	Supporting clinical decision-making and health policy by providing evidence from real-life setting beyond controlled trials
Real-time data (RTD)	Information that is collected and immediately available for use or analysis as events occur, without significant delay	Monitoring devices, clinical care systems	Immediate, as events occur	Required in scenarios requiring immediate action, like critical care monitoring.
Real-world/time data and evidence (RWTD/E)	Conceptualized as combining real-world context with the immediacy of real-time data collection and analysis	EHRs, patient devices, and real-time monitoring systems	Real-time, as events occur in real-world settings	Enhancing understanding of healthcare interventions in real-world settings with the immediacy of real-time data capture

## Data Availability

The data presented in this study are available on request from the corresponding author.

## Appendix A. The SSN-Minimal Data Set (SSN-MDS) Includes the Basic Parameters of Sarcoma Patient Care, Which Allow Data Harmonization over the Geography and Across Institutions

**Institution**	free field
**Pat ID**	number
**Gender (M/F)**	[0] male [1] female
**Date of birth**	DD/MM/YY
**Affected tissue**	[0] Superficial Soft Tissue [1] Deep Soft Tissue (according to the investing fascia; note that a tumor located superficially but invading the investing fascia should be recorded as deep) [2] Bone
**Site of lesion**	[0] Foot [1] Distal lower limb below knee—anterior compartment [2] Distal lower limb below knee—lateral compartment [3] Distal lower limb below knee—posterior compartment [4] Distal lower limb below knee—more than one compartment [5] Popliteal fossa/knee [6] Proximal lower limb at or above knee—anterior compartment [7] Proximal lower limb at or above knee—posterior compartment [8] Proximal lower limb at or above knee—more than one compartment [9] Buttocks and inguinal region [10] Hand [11] Distal upper limb below elbow—ventral compartment [12] Distal upper limb below elbow—dorsal compartment [13] Distal upper limb below elbow—more than one compartment [14] Elbow [15] Proximal upper limb at or above the elbow—ventral compartment [16] Proximal upper limb at or above the elbow—dorsal compartment [17] Proximal upper limb at or above the elbow—more than one compartment [18] Axilla/scapula [19] Head and neck [20] Chest wall [21] Abdominal wall [22] Paraspinal [23] Retroperitoneal [24] Uterus [25] Other visceral [26] Intrathoracic [0] C-spine [27] T-spine [28] L-spine [29] Sacrum [30] Scapula [31] Prox humerus [32] Mid humerus [33] Dist humerus [34] Prox forearm [35] Mid forearm [36] Dist forearm [37] Carpus [38] Hand [39] Clavicle [40] Post pelvis [41] Acetabulum [42] Ant pelvis [43] Prox femur [44] Mid femur [45] Dist femur [46] Prox tibia [47] Mid tibia [48] Dist tibia [49] Fibula [50] Foot
**Disease Status @ diagnosis**	[0] Localized disease [1] Oligometastatic disease (5 or less lesions) [2] Polymetastatic disease (6 and more lesions)
**Date of index surgery**	DD/MM/YY
**Type of index surgery**	[0] Previous inadvertent excision; no re-excision [1] Re-excision after previous primary inadequate resection [2] Primary surgery without soft tissue reconstruction [3] Primary surgery with soft tissue reconstruction [4] Primary surgery without bone reconstruction [5] Primary surgery with bone reconstruction [6] Primary surgery with bone and soft tissue reconstruction
**Margin status**	[0] R0 [1] R1 [2] R2 [3] unknown
**Tumor maximal size (cm)**	If patients has undergone preoperative Tx, please record the largest diameter either pre or post the preop therapy
**Date of histological diagnosis**	DD/MM/YY
**Histological diagnosis**	[0] Angiosarcoma of soft tissues [1] Atypical chondromatous tumor [2] Atypical lipomatous tumor/well differentiated liposarcoma [3] Chondrosarcoma [4] Conventional osteosarcoma [5] Dedifferentiated liposarcoma [6] Desmoid-type fibromatosis [7] Ewing sarcoma [8] Giant cell tumor of bone [9] GIST [10] Intramuscular myxoma [incl. variants] [11] Leiomyosarcoma [excluding skin] [12] Myxofibrosarcoma [13] Myxoid liposarcoma [14] Pleomorphic liposarcoma [15] Solitary fibrous tumor [16] Synovial sarcoma [17] Tenosynovial giant cell tumor [18] Undifferentiated/unclassified sarcoma
**If other, please specify**	Free text
**Grading (FNCLCC)**	[0] Grade 1 (Low grade): Total score 3–5 [1] Grade 2 (Intermediate grade): Total score 6–7 [2] Grade 3 (High grade): Total score 8–9 [3] Unknown [4] None available
**Chemotherapy (index)**	[0] No therapy [1] Neoadjuvant (localized disease) [2] Preoperative (metastatic) [3] Adjuvant (localized) [4] Postoperative (metastatic) [5] Palliative intent: treatment pressure
**Radiotherapy (index; type of administration)**	[0] Preoperative [1] Postoperative [2] Pre- and postoperative [3] IORT [4] Brachytherapy [5] Preoperative + IORT [6] Preoperative + brachytherapy [7] IORT + postoperative [8] Brachytherapy + postoperative [9] IORT + postoperative [10] Brachytherapy + postoperative operative [11] In combination with hyperthermia [12] None
**Radiotherapy (index; schedule)**	[0] Normofractionated 25 × 2 Gy [1] Hypofractionated 15 × 3 Gy [2] Ultrahypofractionated 5 × 5 Gy [3] Other
**Date of 1st local recurrence**	DD/MM/YY; otherwise “none”
**Treatment of 1st LR**	[0] Surgery [1] Chemotherapty [2] Radiotherapy [3] Surgery + Radiotherapy [4] Surgery + Chemotherapty [5] Surgery + Chemotherapty + Radiotherapy [7] Radiotherapy + Chemotherapty [6] Best supportive care
**Date of 1st LR surgery**	DD/MM/YY
**Date of 2nd LR**	DD/MM/YY
**Treatment of 2nd LR**	[0] Surgery [1] Chemotherapty [2] Radiotherapy [3] Surgery + Radiotherapy [4] Surgery + Chemotherapty [5] Surgery + Chemotherapty + Radiotherapy [7] Radiotherapy + Chemotherapty [6] Best supportive care
**Date of 2nd LR surgery**	DD/MM/YY
**Date of pulmonary metastasis**	DD/MM/YY; otherwise “none”
**Date of extrapulmonary metastasis**	DD/MM/YY; otherwise “none”
**Site of extrapulmonary metastasis**	free field
**Treatment of metastasis**	[0] Surgery [1] Chemotherapty [2] Radiotherapy [3] Surgery + Radiotherapy [4] Surgery + Chemotherapty [5] Surgery + Chemotherapty + Radiotherapy [7] Radiotherapy + Chemotherapty [6] Best supportive care
**Date of last follow-up**	DD/MM/YY
**Status**	[0] NED (no evidence of disease) [1] AWD (alive with disease) [2] DOD (dead of disease) [3] DOC (dead of other causes)
